# Role of cerebral hypoperfusion in multiple sclerosis (ROCHIMS): study protocol for a proof-of-concept randomized controlled trial with bosentan

**DOI:** 10.1186/s13063-019-3252-4

**Published:** 2019-03-14

**Authors:** Stéphanie Hostenbach, Ayla Pauwels, Veronique Michiels, Hubert Raeymaekers, Anne-Marie Van Binst, Annick Van Merhaeghen-Wieleman, Peter Van Schuerbeek, Jacques De Keyser, Miguel D’Haeseleer

**Affiliations:** 1 Department of Neurology, Universitair Ziekenhuis (UZ) Brussel, Brussels, Belgium; 2 Center for Neurosciences, Vrije Universiteit Brussel, Brussels, Belgium; 3 Department of Radiology and Medical Physics, UZ Brussel, Brussels, Belgium; 4 Department of Neurology, Universitair Medisch Centrum Groningen, Groningen, The Netherlands; 5 National Multiple Sclerosis Centrum, Melsbroek, Belgium

**Keywords:** Multiple sclerosis, Cerebral blood flow, N-acetyl aspartate, Bosentan, Axonal degeneration

## Abstract

**Background:**

Axonal degeneration is related to long-term disability in patients with multiple sclerosis (MS). The underlying mechanism remains ill understood but appears to involve axonal energetic dysfunction. A globally impaired cerebral blood flow (CBF) has been observed in the normal-appearing white matter (NAWM) of patients with MS, which is probably related to astrocytic overexpression of endothelin-1 (ET-1). Cerebral hypoperfusion has been associated with reduced mitochondrial activity and disabling symptoms (e.g. fatigue and cognitive decline) of MS. Countering this process could therefore be beneficial in the disease course. Short-term CBF restoration with a single 62.5-mg dose of the ET-1 receptor antagonist bosentan has already been demonstrated in patients with MS.

**Methods:**

The ROCHIMS study is a proof-of-concept double-blind randomized clinical trial in which patients with relapsing-remitting MS will receive either 62.5 mg bosentan or matching placebo twice daily during 28 ± 2 days. Clinical evaluation and brain magnetic resonance imaging (MRI) will be performed at baseline and treatment termination. Based on previous work, we expect a global increase of CBF in the individuals treated with bosentan. The primary outcome measure is the change of N-acetyl aspartate in centrum semiovale NAWM, which is a marker of regional axonal mitochondrial activity. Other parameters of interest include changes in fatigue, cognition, motor function, depression, and brain volume.

**Discussion:**

We hypothesize that restoring cerebral hypoperfusion in MS patients improves axonal metabolism.

Early positive effects on fatigue and cognitive dysfunction related to MS might additionally be detected. There is a medical need for drugs that can slow down the progressive axonal degeneration in MS, making this an important topic of interest.

**Trial registration:**

Clinical Trials Register, EudraCT 2017-001253-13. Registered on 15 February 2018.

**Electronic supplementary material:**

The online version of this article (10.1186/s13063-019-3252-4) contains supplementary material, which is available to authorized users.

## Background

Multiple sclerosis (MS) is a chronic neurodegenerative and inflammatory disorder of the central nerve system (CNS) mainly affecting young adults [1]. Disease pathology is characterized by two key players: intermittent bursts of focal inflammatory demyelination and widespread progressive axonal degeneration. The clinical course is highly variable. Most individuals start with a relapsing-remitting pattern, in which newly formed clinical deficits restore partially or completely over time, but after several years they often develop a secondary progressive phase of slower but continuous neurological worsening. Other patients experience primary progressive disease from onset [2].

MS relapses are triggered by the formation of focal inflammatory lesions in which auto-immune reactions against CNS myelin play a paramount role. In contrast, the steadier decline mainly results from progressive axonal loss [3]. The exact underlying mechanism of the latter remains unclear and degenerative features do not seem to correlate well with inflammatory activity, particularly in the later stages of the disease [4]. Recognition is growing that mitochondrial energy failure and oxidative stress are involved in this axonal degeneration process of MS [5, 6]. Levels of N-acetylasparate (NAA), an amino acid synthesized by mitochondria in neurons and their axons, are a marker of both axonal mitochondrial activity and axonal integrity [7] and were found to be reduced in several ^1^H-magnetic resonance spectroscopy (MRS) studies of centrum semiovale normal-appearing white matter (NAWM) in patients with MS compared to controls [8].

Perfusion-weighted magnetic resonance imaging (MRI) research conducted over the past decade has found that cerebral blood flow (CBF) is globally impaired in the NAWM of patients with relapsing-remitting MS (RRMS), primary progressive MS, and even clinically isolated syndromes suggestive for MS (CIS-MS) [9–11]. Similar observations have been made in the thalamus of CIS-MS and RRMS individuals [12]. These findings suggest that cerebral hypoperfusion is an early and integral feature of MS pathology, regardless of the clinical subtype [5]. It is unknown whether this phenomenon contributes to the progressive loss of axons, but chronically induced cerebral hypoperfusion in animal models leads to mitochondrial energy failure, oxidative stress, and eventually axonal degeneration [13]. In addition, reduced CBF has been associated with cognitive dysfunction and fatigue in patients with MS [14], which are both disabling but poorly understood disease manifestations. Reduced cerebral perfusion in small rodents resulted in neuronal loss in the hippocampal CA1 sub region, while MRI studies in MS patients revealed selective and progressive hippocampal atrophy localized initially to this CA1 sub region, which may contribute, among other factors, to cognitive impairment that occurs in many of these individuals [15, 16].

Reduced CBF in MS appears to be mediated by elevated levels of the potent vasoconstrictive agent endothelin-1 (ET-1) in the cerebral circulation, most likely derived from reactive astrocytes in focal lesions. In a previous study, the administration of a single 62.5 mg dose of bosentan, which is an ET-1 receptor antagonist, increased CBF in patients with MS up to the levels obtained in controls [5]. The main goal of the present study is to investigate in NAWM of patients with RRMS whether prolonged treatment with bosentan results in a sustained restoration of CBF and whether this enhances NAA levels, reflecting improvement of axonal mitochondrial metabolism. Our secondary aim is to explore whether CBF restoration ameliorates clinical disability (fatigue, cognition, motor function, and depression) and hippocampal volume in these patients.

## Methods and design

### Trial design

This study is a non-commercial proof-of-concept, placebo-controlled, and double-blind randomized trial, taking place at the Universitair Ziekenhuis (UZ) Brussel between October 2017 and December 2018. It is approved at national level by the Federaal Agentschap Voor Geneesmiddelen en Gezondheidsproducten (FAGG) and the local ethics committee of the UZ Brussel. The study is registered by the European Union Drug Regulating Authorities (EudraCT 2017-001253-13) as the ROCHIMS (Role Of Cerebral Hypoperfusion In Multiple Sclerosis) trial. In case of protocol modifications, all relevant stakeholders will be informed.

### Eligibility criteria

All potential participants will be screened for study inclusion according to the criteria outlined below. Each patient will be informed about the study protocol by the principle investigator and written informed consent will be obtained from all participants.

#### Inclusion criteria

- Age > 18 years;
- Diagnosis of relapsing-remitting MS, according to the 2010 revised McDonald criteria [17];
- Expanded Disability Scale Score (EDSS) ≤ 4.0 [18];
- Use of reliable means of contraception for sexually active female patients.

#### Exclusion criteria

- Clinical evidence of MS relapse within the last three months before inclusion;
- Pregnant or lactating women;
- Major liver dysfunction (AST and/or ALT > 3 times ULN);
- Use of cyclosporine A or glibenclamide;
- Allergy to bosentan.

### Randomization, study medication, and general outline

Participants will be randomly divided into a group receiving bosentan (Actelion Pharmaceuticals Ltd., Allschwil, Switzerland) 62.5 mg twice daily and another group receiving matching placebo, during 28 (± 2) days. The randomization will be performed with “research randomizer” (https://randomizer.org), using five sets of six numbers in order to achieve an equal distribution between active product and placebo. Study medication will be stored at the Hospital Pharmacy. After inclusion, patients are given a computer-generated randomization number, which is matched to a confidential treatment number by the responsible hospital pharmacist in order to assign participants to either bosentan or placebo.

Bosentan tablets are provided by Actelion Pharmaceuticals Belgium NV (Mechelen, Belgium). The production of identical-looking placebo tablets is performed by the hospital pharmacy. The tablets are taken twice daily, at 08:00 and 20:00. Adherence to the study medication will be assessed by counting the number of unused tablets returned at the second visit. All adverse events will be recorded in the “Adverse Event” section of the Case Report Form. All methods for data collection and data management will be collected in the Investigator Site File. The data monitoring committee of this trial is the Clinical Trial Centre of the University Hospital Brussel. This Clinical Trial Centre works independent from the sponsor and have no competing interests.

**Fig. 1:**
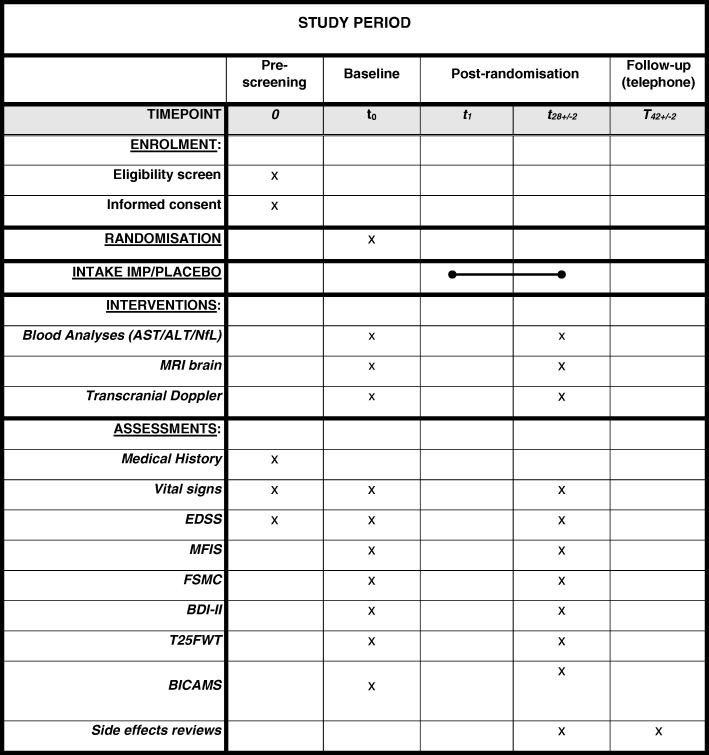
ROCHIMS Trial time table. SPIRIT Figure. (See also list of abbreviations)

At baseline (day 0), patients will undergo a blood analysis, clinical assessments, transcranial Doppler (TCD), and brain MRI. All data will be pseudonymized. Only the principle investigator has the key to source data. Treatment will commence the next day (day 1). On day 28 (± 2 days), all evaluations will be repeated. All tests will be performed between 09:00 and 13:00 to avoid interference of fatigue accumulation during the day with the results. Participants are asked to refrain from alcohol or caffeine, as well as not to smoke, on the evaluation days. Two weeks after ending the trial, participants will be called to see whether any side effects has occurred in that period. After closure of the study and unblinding, only the individual patients will be informed about their study medication and all biological specimens will be destroyed. Communication of the study results will be anonymized. The trial time table is presented in Fig. 1 (Additional file 1). The study protocol is constituted taking into account the SPIRIT guidelines; the SPRIT checklist can be found as an attachment of this manuscript.

### Definition of outcome measures

#### Primary outcome

The primary outcome measure of the study is the change in NAA/creatine (NAA/Cr) ratios in centrum semiovale NAWM, measured with ^1^H-MRS as a part of the MRI protocol, reflecting changes in axonal mitochondrial metabolism. Based on our previous findings, we expect a rise of CBF values after treatment with bosentan [5]. The MRI protocol contains a pseudo-continuous arterial spin labeling (ASL) sequence to assess CBF in the NAWM of the centrum semiovale.

#### Secondary outcomes

1. Changes in fatigue, cognition, symptoms of depression, and motor function.

Clinical assessments will in each individual be performed by the same trained MS study nurse or neurologist in training to ensure consistency. They were trained in advance by an internal senior staff member to perform the neuropsychological assessments. Assessments are always scheduled in the same order: fatigue and depression scales and cognitive testing before motor function testing to avoid the interference of physical fatigue with the results. The assessments include the following:

1.1. * Fatigue scales*: Fatigue Severity Scale (FSS) [19], Modified Fatigue Impact Scale – 5-item version (MFIS-5) [20], and Fatigue Scale for Motor and Cognitive functions (FSMC) [21]. These scales assess the experienced severity of fatigue symptoms and/or the impact on cognitive, physical, and psychosocial functioning in the preceding four weeks.
1.2. *Depressive symptomatology scales*: Beck Depression Inventory (BDI-II), which is a widely used to assess the presence of symptoms of depression in the two weeks preceding the questionnaire [22].
1.3. *Cognitive performance scales*: Brief International Cognitive Assessment for Multiple Sclerosis (BICAMS): SDMT (Symbol Digit Modality Test), CVLT-II (California Verbal Learning Test), and BVMT-R (Brief Visuospatial Memory Test-Revised). The BICAMS is considered a brief and feasible tool appropriate for the cognitive assessment of MS patients [23]. The SDMT is a test of information processing speed with a high sensitivity to cognitive impairment in MS, the CVLT-II is a test for short-term and long-term verbal memory, and the BVMT-R evaluates visuospatial memory abilities [24].
1.4. *Motor function testing*: Timed 25 Foot Walk Test (T25FWT) and 9-Hole Peg Test (9HPT). T25FWT is a quantitative mobility and leg function performance test based on a timed 25-ft (or 7.62 m) walk. The patient is instructed to walk as quick as possible, without running, and safely. The time needed to perform this task, starts from the instruction to start and ends when the patient reach the 25-ft mark. Afterwards, the patient will walk the same distance back. The average of these two times is the score for the T25FWT. The 9-HPT is a quantitative measure of upper extremity (arm and hand) function. Patients have to pick up nine little pegs one by one from a container, put them as quickly as possible in nine empty holes, and once they are all in, the patients have to replace them in the container one by one. The total time for this is measured. Patients have to perform this task twice with both hands. The total score of the 9-HPT is the average time of the four trials [25].

2. Brain volumetric changes will be investigated by means of voxel-based morphometry (VBM) analysis, in which baseline T1-weighted images are subtracted from analogue follow-up images, to obtain images that represent changes of time for each individual patient. Both hippocampal regions will be selected as the main ROI for VBM analysis.
3. Changes in serum levels of light chain neurofilament (NfL).

Axonal damage has been associated with a release of NfL, which is a compound of the axonal cytoskeleton, into the cerebrospinal fluid (CSF) and peripheral blood of patients with MS. New high-sensitive techniques allow the detection of NfL differences at low concentration in the serum, which has the advantage of being more easily accessible as CSF [26, 27].

4. Changes in cerebral hemodynamic parameters.

Cerebral circulation will additionally be assessed by means of TCD, which is an easily applicable bedside tool, for explorative reasons.

### MRI protocol

All patients undergo a multimodal imaging protocol consisting of a high-resolution anatomical scan (T1 3D BRAVO, FOV = 256 × 256 × 180 mm^3^, resolution = 1 × 1 × 1 mm^3^, TR = 8.5 ms, TE = 3.2 ms, flip angle = 12°, TI = 450 ms; sagittal scan orientation, axial and coronal reconstructions were made afterwards), 3D fluid-attenuated inversion recovery (3D Cube T2 FLAIR, FOV = 256 × 256 × 180 mm^3^, resolution = 1 × 1 × 1 mm^3^, TR = 8000 ms, TE = 130 ms, flip angle = 90°; sagittal scan orientation, axial and coronal reconstructions were made afterwards) imaging, ASL (3D ASL, FOV = 240 × 240 mm^3^, spiral scan with 8 arms and 512 point per arm, 36 slices, slice thickness = 4.0 mm, TR = 4852 ms, TE = 10.7 ms, flip angle = 90°, TI = 2025 ms; axial plane orientation) and ^1^H-MRS (MRS PROBE, FOV = 20 × 20 mm^2^, slice thickness = 10 mm, 12 × 12 voxels, voxel thickness optimized per individual, TR = 2000 ms, TE = 144 ms, NEX = 1.6) on a 3-T machine (GE, MR 750 W Discovery).

The regions of interest for CBF and NAA/Cr + PCr quantification are the left and right centrum semiovale NAWM. FLAIR images are used to avoid inclusion of focal MS plaques. Brain volumetric changes after treatment are investigated by VBM analysis using Statistical Parametric Mapping 8 software. Total scanning time takes about 30 min.

For anatomical reference, the axial reconstructed T1-weighted BRAVO images were used; an EPISTAR (echo-planar imaging and signal targeting with alternating radio-frequency) sequence is used. The slice orientation is transversal and imaging volume is centered on the centrum semiovale. An EPI sequence with the same parameters and orientation, but without labelling pulses, is used to measure equilibrium magnetization. CBF is calculated in the NAWM of the right and left centrum semiovale by using a FLAIR scan to avoid placement in plaques. Longitudinal relaxation rates for tissue (R1app) was 0.91 s-1 and for blood (R1b) 0,59 s-1. The equilibrium magnetization of the white matter (M0WM) is used to determinate the equilibrium magnetization of blood (M0B). T2 relaxation times for white matter and blood are 55 ms and 100 ms, respectively, which brings us to the following relationship: M0B = 1.28 M0WM [10]. The final CBF results represent the average of the left and right centrum semiovale.

NAA is expressed as ratios to total creatinine (Cr, sum of Cr, and PCr), measured by ^1^H-MRS. Total Cr is supposed to maintain a relatively constant level [28]. A two-dimensional (2D) spectroscopic imaging slab, which contains multiple voxels of 1.5 cm^3^, is positioned in the centrum semiovale above the corpus callosum. Water suppressed single-slice-point-resolved spectroscopy is used with the following scan parameters: TE = 144 ms; TR = 2 s; slice thickness = 1.5 cm; in-plane resolution = 1 × 1 cm^2^; spectral band width = 2000 Hz, and 1024 sample points. NAA and Cr levels are analyzed in different voxels in the NAWM of the centrum semiovale of both cerebral hemispheres. Voxels including plaques and black holes are excluded. Analysis of the spectroscopic data is performed using GE Functool software, that allows operator-independent quantification of NAA, Cr + PCr, and NAA/Cr + PCr ratios.

### Blinding and statistical methods

Sample size calculation is based on the study of Steen et al. where NAA/Cr in centrum semiovale NAWM was 10% lower in MS patients compared to healthy controls [8]. The inclusion of 24 individuals has 90% power to detect a 10% increase in NAA/Cr in centrum semiovale NAWM in the bosentan group compared to the placebo group, with a significance level of 0.05. Taking into account asymptomatic efficiency loss (non-parametric study of 5%) and potential drop outs of 5%, inclusion of 30 patients should be sufficient.

All study participants and investigators will remain blinded throughout the complete treatment and evaluation period. Unblinding will only be done after final database lock or in case of emergencies and serious adverse events.

All statistics will be performed by an independent biometrician and with SPSS software, version 25. The Wilcoxon signed-rank test for paired samples will be used to compare differences within the two treatment groups; the Mann–Whitney U test will be used to compare differences between the groups. Fisher’s exact test will be used to compare categorical variables. Spearman’s correlation coefficient will be used to correlate continuous data. The level for statistical significance is defined as *p* < 0.05.

## Discussion

Cerebral perfusion appears to be unequivocally and globally decreased in all subtypes of MS. Pathological repercussions have hitherto received little attention but might include fatigue, cognitive impairment, and contribution to the hallmark process of axonal degeneration, possibly by compromising mitochondrial energy production. In a previous study, a single dose of 62.5 mg bosentan was able to restore reduced CBF in patients with MS up to the levels measured in healthy controls, revealing a potential new therapeutic target [5]. The ROCHIMS study is the first proof-of-concept clinical trial evaluating the effects of CBF restoration in patients with MS over a longer treatment period. We hypothesize that countering cerebral hypoperfusion in MS patients for one month improves axonal metabolism, as reflected by NAA levels measured with ^1^H-MRS. Beneficial effects may also be noticeable on clinical (fatigue, cognition, motor function, depression) and other imaging outcome parameters (hippocampal volume).

There is a medical need for drugs that can halt or slow down progressive axonal degeneration in MS. Repurposing drugs for proof-of-concept trials in this indication is therefore of considerable interest. Bosentan is already widely available for other medical conditions than MS and the side effects, which in general are rather mild, are well known. A limitation of this trial is the short treatment period. The results of this proof-of-concept trial will form the basis for further trials with a longer period of treatment and more focused on clinical outcome measures, especially progression of disability.

## Trial status

The ROCHIMS study is currently recruiting patients. The protocol “Versie 2.0 ROCHIMS” dated 16 August 2017 was approved by the FAGG and the local ethics committee of the UZ Brussel. We started recruiting on the first of September 2017 and will approximately end the recruitment in April 2019.

## Additional file

Additional file 1 : SPIRIT 2013 Checklist: Recommended items to address in a clinical trial protocol and related documents. (DOC 122 kb)

## Abbreviations

9HPT: 9-Hole Peg Test
ALT: Alanine aminotransferase
ASL: Arterial spin labeling
AST: Aspartate aminotransferase
BBB: Blood–brain barrier
BDI-II: Beck Depression Inventory
BICAMS: Brief International Cognitive Assessment for Multiple Sclerosis
BVMT-R: Brief Visuospatial Memory Test-Revised
CBF: Cerebral blood flow
CIS-MS: Isolated syndromes suggestive for MS
Cr: Creatinine
CSF: Cerebrospinal fluid
CVLT-II: California verbal learning test
EDSS: Expanded disability scale score
EPISTAR: Echo-planar imaging and signal targeting with alternating radio-frequency
ET-1: Endothelin-1
FAGG: “Federaal Agentschap Voor Geneesmiddelen en Gezondheidsproducten”
FLAIR: Fluid-attenuated inversion recovery
FOV: Field of view
FSMC: Fatigue Scale for Motor and Cognitive functions
FSS: Fatigue severity scale
HIF-1: Hypoxia-inducible factor 1
MFIS-5: Modified fatigue impact Scale 5-item version
MRS: 1H-MR spectroscopy
MS: Multiple sclerosis
NAA: N-acetylaspartate
NAWM: Normal-appearing white matter
NfL: Serum neurofilament light chains
ROCHIMS: Role of cerebral hypoperfusion in MS patients
RRMS: Relapsing-remitting MS
SDMT: Symbol Digit Modality Test
T25FWT: Timed 25 Foot Walk Test
TCD: Transcranial Doppler
ULN: Upper limits of normal
VBM: Voxel-based morphometry

## References

[1] Liguori M, Marrosu MG, Pugliatti M, Giuliani F, De Robertis F, Cocco E (2000). Age at onset in multiple sclerosis. Neurol Sci.

[2] Cambron M, D'Haeseleer M, Laureys G, Clinckers R, Debruyne J, De Keyser J (2012). White-matter astrocytes, axonal energy metabolism, and axonal degeneration in multiple sclerosis. J Cereb Blood Flow Metab.

[3] Evangelou N, Esiri MM, Smith S, Palace J, Matthews PM (2000). Quantitative pathological evidence for axonal loss in normal appearing white matter in multiple sclerosis. Ann Neurol.

[4] Tremlett H, Yousefi M, Devonshire V, Rieckmann P, Zhao Y, Neurologists U (2009). Impact of multiple sclerosis relapses on progression diminishes with time. Neurology.

[5] D'haeseleer M, Beelen R, Fierens Y, Cambron M, Vanbinst AM, Verborgh C (2013). Cerebral hypoperfusion in multiple sclerosis is reversible and mediated by endothelin-1. Proc Natl Acad Sci U S A.

[6] Trapp BD, Stys PK (2009). Virtual hypoxia and chronic necrosis of demyelinated axons in multiple sclerosis. Lancet Neurol.

[7] Moffett JR, Ross B, Arun P, Madhavarao CN, Namboodiri AM (2007). N-Acetylaspartate in the CNS: from neurodiagnostics to neurobiology. Prog Neurobiol.

[8] Steen C, D'haeseleer M, Hoogduin JM, Fierens Y, Cambron M, Mostert JP (2013). Cerebral white matter blood flow and energy metabolism in multiple sclerosis. Mult Scler.

[9] Law M, Saindane AM, Ge Y, Babb JS, Johnson G, Mannon LJ (2004). Microvascular abnormality in relapsing-remitting multiple sclerosis: perfusion MR imaging findings in normal-appearing white matter. Radiology.

[10] Adhya S, Johnson G, Herbert J, Jaggi H, Babb JS, Grossman RI (2006). Pattern of hemodynamic impairment in multiple sclerosis: dynamic susceptibility contrast perfusion MR imaging at 3.0 T. Neuroimage.

[11] Varga AW, Johnson G, Babb JS, Herbert J, Grossman RI, Inglese M (2009). White matter hemodynamic abnormalities precede sub-cortical gray matter changes in multiple sclerosis. J Neurol Sci.

[12] Papadaki EZ, Mastorodemos VC, Amanakis EZ, Tsekouras KC, Papadakis AE, Tsavalas ND (2012). White matter and deep gray matter hemodynamic changes in multiple sclerosis patients with clinically isolated syndrome. Magn Reson Med.

[13] Aliev G, Smith MA, Obrenovich ME, de la Torre JC, Perry G (2003). Role of vascular hypoperfusion-induced oxidative stress and mitochondria failure in the pathogenesis of Azheimer disease. Neurotox Res.

[14] D'haeseleer M, Hostenbach S, Peeters I, Sankari SE, Nagels G, De Keyser J (2015). Cerebral hypoperfusion: a new pathophysiologic concept in multiple sclerosis?. J Cereb Blood Flow Metab.

[15] Chiaravalloti ND, DeLuca J (2008). Cognitive impairment in multiple sclerosis. Lancet Neurol.

[16] Sicotte NL, Kern KC, Giesser BS, Arshanapalli A, Schultz A, Montag M (2008). Regional hippocampal atrophy in multiple sclerosis. Brain.

[17] Milo R, Miller A (2014). Revised diagnostic criteria of multiple sclerosis. Autoimmun Rev.

[18] Kurtzke JF (1983). Rating neurologic impairment in multiple sclerosis: an expanded disability status scale (EDSS). Neurology.

[19] Krupp LB, LaRocca NG, Muir-Nash J, Steinberg AD (1989). The fatigue severity scale. Application to patients with multiple sclerosis and systemic lupus erythematosus. Arch Neurol.

[20] D’Souza E (2016). Modified fatigue impact scale – 5-item version (MFIS-5). Occup Med.

[21] Dittner AJ, Wessely SC, Brown RG (2004). The assessment of fatigue: a practical guide for clinicians and researchers. J Psychosom Res.

[22] Wang YP, Gorenstein C (2013). Psychometric properties of the Beck Depression Inventory-II: a comprehensive review. Rev Bras Psiquiatr.

[23] Niccolai C, Portaccio E, Goretti B, Hakiki B, Giannini M, Pastò L (2015). A comparison of the brief international cognitive assessment for multiple sclerosis and the brief repeatable battery in multiple sclerosis patients. BMC Neurol.

[24] Langdon DW, Amato MP, Boringa J, Brochet B, Foley F, Fredrikson S (2012). Recommendations for a Brief International Cognitive Assessment for Multiple Sclerosis (BICAMS). Mult Scler.

[25] Fischer JS, Jak AJ, Kniker JE, Rudick RA, Cutter G. Multiple Sclerosis functional composite (MSFC): administration and scoring manual: National Multiple Sclerosis Society; 2001. http://main.nationalmssociety.org/docs/HOM/MSFC_Manual_and_Forms.pdf.

[26] Brureau A, Blanchard-Bregeon V, Pech C, Hamon S, Chaillou P, Guillemot JC (2017). NF-L in cerebrospinal fluid and serum is a biomarker of neuronal damage in an inducible mouse model of neurodegeneration. Neurobiol Dis.

[27] Kuhle J, Barro C, Disanto G, Mathias A, Soneson C, Bonnier G (2016). Serum neurofilament light chain in early relapsing remitting MS is increased and correlates with CSF levels and with MRI measures of disease severity. Mult Scler.

[28] Graumann U, Reynolds R, Steck AJ, Schaeren-Wiemers N (2003). Molecular changes in normal appearing white matter in multiple sclerosis are characteristic of neuroprotective mechanisms against hypoxic insult. Brain Pathol.

